# Gills Just Want to Have Fun: Can Fish Play Games, Just like Us?

**DOI:** 10.3390/ani12131684

**Published:** 2022-06-30

**Authors:** Sofia Eisenbeiser, Étienne Serbe-Kamp, Gregory J. Gage, Timothy C. Marzullo

**Affiliations:** 1 Research and Development, Backyard Brains, Ann Arbor, MI 48104, USA; seisenb3@emich.edu (S.E.); etienne@backyardbrains.com (É.S.-K.); gagegreg@backyardbrains.com (G.J.G.); 2 Neuroscience Department, Eastern Michigan University, Ypsilanti, MI 48197, USA; 3 Hirnkastl, Lindwurmstraße 21, 80337 München, Germany; 4 Department of Circuits-Computation-Models, Max Planck Institute of Neurobiology, 82152 München, Germany

**Keywords:** fish behavior, play, cognitive development, comparative biology

## Abstract

**Simple Summary:**

A pending question in animal biology is whether fish are capable of complex behaviors, such as play. We investigated this by shining laser pointers of various colors into home fish tank aquariums. We tested 66 different species and found that over 80% of fish showed an inquisitive response to the moving light stimuli, with the greatest interest in red laser spots. We review the literature on fish play and discuss whether the fish responses we observed can be considered play.

**Abstract:**

It is common to observe play in dogs, cats, and birds, but have we been ignoring play in one of the most common house pets of all… fish? Aquarium fish are often used as meditative decoration in family households, but it could be that fish have similarly diverse behavioral repertoires as mammals and birds. To examine this theory, we conducted field tests at local pet stores where a range of aquarium fish species was tested for responsiveness to laser pointer stimuli. Out of 66 species of fish tested, over 80% showed a tendency to be interested in the moving laser spots, particularly red ones. Whether this behavior constitutes play is an active topic of investigation that we examine in this work.

## 1. Introduction

Accounts of play behaviors in fish exist but are not widespread in the scientific literature. One 2015 study by Burghardt et al. [1] determined that three white-spotted African cichlids (*Tropheus duboisi*) exhibited play behavior with self-righting thermometers by repeatedly trying to knock them down and move them around in their respective aquariums. Gunter [2] and Ladiges [3] also reported similarly unusual behaviors in fishes, citing rough silverside (*Membras vagrans*) fish who swam around, head-butted, and charged a nylon line, along with a sterlet (*Acipenser ruthenus*) who would vigorously push and pull objects in its tank. Elephant fish (*Mormyrus*), a family of fish with very large brains for their size [4], have been observed playing with twigs, pushing them around the aquarium and on top of their snouts even when not hungry [5] and will manipulate plastic balls around their tanks [6]. Tool use, another cognitively complex behavior, has been observed in wrasses (*Labridae*) and tuskfish (*Choerodon schoenleinii*), who crush mollusks in their mouths against rocks to break them open [7,8].

Other notable reports of fish behavior—particularly regarding their varied responses to artificial light stimuli, include a recent study by Cohn et al. [9], who showed that particular anemonefish (*Amphiprion chrysopterus*) and damselfish (*Dascyllus trimaculatus*) species, residing in a magnificent anemone (*Heteractis magnifica*) off of the French Polynesian coast island of Mo’orea, lunged at a red laser stimulus that the investigators circled around the anemone. This behavior was interpreted as a territory threat response rather than a prey response, as there was no attempt by the fishes to bite the laser. Diverse responses to laser stimuli have also been observed in wrasse (*Labridea*) and other species as part of coastal health surveying missions off the southern coasts of England and British Columbia [10,11]. The projection of images onto the sides of laboratory fish tanks was used as a technique to study predation behavior in bluegill sunfish (*Lepomis macrochirus*) [12], and attention and facial recognition in the lyretail cichlid (*Neolamprologus brichardi*) was examined using photos accompanied by red laser points [13].

Fish can display a diverse behavioral repertoire, but can we reasonably and scientifically define some behaviors as play? One potential solution comes from Burghardt [14], who proposed a set of five criteria for classifying play in animals:

1. The behavior is “incompletely functional” and does not contribute to immediate survival.
2. The behavior is voluntary, spontaneous, intentional, and performed for its own sake.
3. The behavior may resemble completely functional behaviors, but it differs in at least one respect, such as context, or is somehow incomplete, exaggerated, or awkward.
4. The behavior is repeated consistently during at least a portion of the animal’s life but is not pathological.
5. The behavior is begun in the absence of stress, hunger, predation, or circumstances that are otherwise unhealthy.

Using these criteria and some creativity, scientists of all ages can dive into the scientific riddles of play and animal cognition. In this article, we detail an easily replicable experiment utilizing laser pointers and aquarium fishes that examines the compelling idea that perhaps fish do, indeed, engage in play.

## 2. Materials and Methods

We brought red, green, and blue standard laser pointers (typically sold as cat toys, purchased from Amazon: ASIN B099DPGJXM—Interactive Cat Toy for Kitten and Dogs 3Pack) to local pet stores and shined each into available aquariums to observe the fishes’ responses and behavioral changes upon the presentation of the laser stimuli. Care was taken to avoid their eyes. Permission from the pet store employees was asked prior to any observations.

Changes in behavior (i.e., interest in laser stimuli) were classified into four response categories: “none”, “some”, “moderate” and “high”. “None” indicated no behavioral changes upon presentation of laser stimulus; “some” indicated a small change in behavior, recognized as a very brief change in swimming direction or orienting toward the laser; “moderate” indicate that fish would show interest in the stimulus for up to roughly five seconds by following and investigating it; and “high” indicated great levels of behavioral changes in which the fish would interact with the laser stimulus for five or more seconds. 

**Figure 1:**
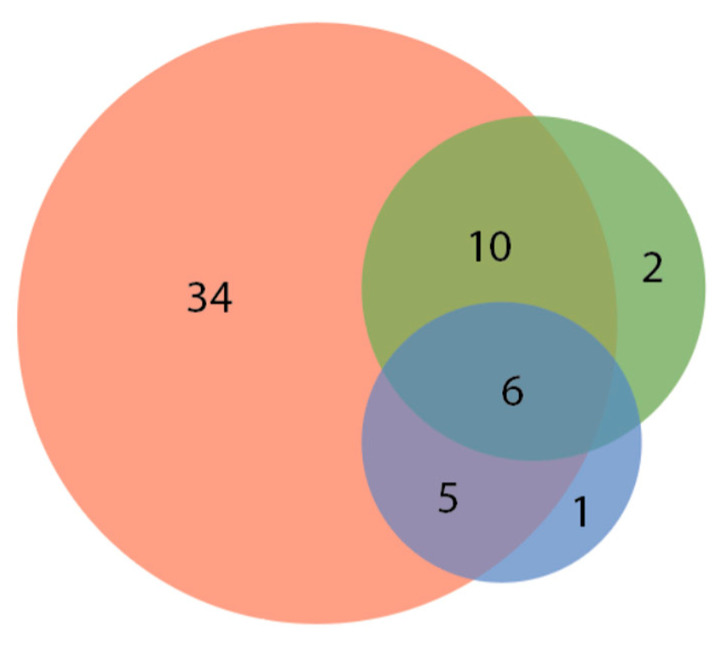
Preference of all tested fish species for laser pointer dot color, with 34 species preferring only red, 2 preferring only green, 1 preferring only blue, 10 preferring red and green, 5 preferring red and blue, and 6 preferring all three colors. No species appeared to only prefer blue and green. (All 8 species in “None” category not represented. See Table 1).

A total of seven trips were made to three different pet stores, all during the mid-afternoon, and we recorded in our lab notebooks the fishes’ degrees of interest in laser pointer dots and laser color preference (Figure 1, Table 1). Author S.E. collected the data on all seven pet store visits and conducted all the behavioral scoring, with authors É.S.-K. and T.C.M. assisting with observations in the home lab and during one pet store visit. During another visit, we purchased eight highly responsive tiger barbs (*Puntigrus tetrazona*) to continuously observe over a period of five weeks. Additionally, we repeatedly investigated laser stimulus responses and self-righting thermometer play in three male white-spotted African cichlids [1] in our home lab over the course of ten weeks. Other than the three male cichlids, the sexes of all other fish studied were unknown.

The fish in the pet stores were typically stored in 15–240-gallon aquariums with 15–100 fish of various species inside. The white-spotted cichlids in our home lab were stored in one 35-gallon tank with equally spaced partitions for the three male fish. The tiger barbs in our home lab were housed together in a 15-gallon tank.

Similar to work by other groups [15,16] in which video recordings of animals in aquariums were analyzed and classified into distinct behaviors to generate ethograms and/or create a catalog of behaviors [17], we analyzed video recordings of the one white-spotted cichlid in our home aquarium that was responsive to the laser stimulus (Supplementary Materials File S2). This analysis isolated behaviors that could be observed during free-swimming (times during which the laser stimulus was not being presented) and laser stimulus presentation epochs, and we tallied each time those behaviors occurred. The behaviors were equally weighted on the basis of the frequency of occurrence and duration.

**Figure 2:**
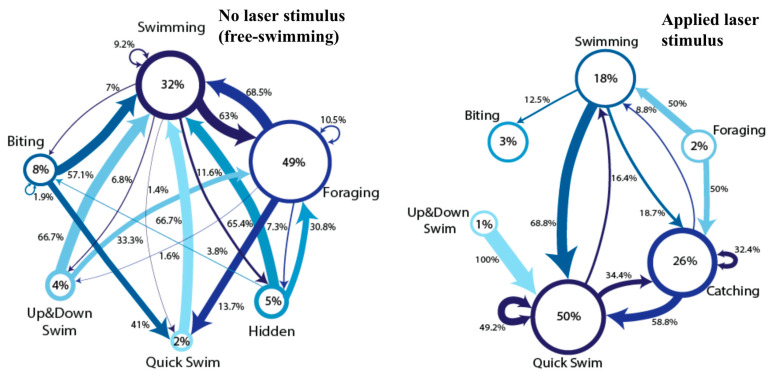
Comparative ethograms of a white-spotted cichlid fish with (**left**) and without (**right**) presentation of red laser stimulus. Diameter of circles corresponds to how much time was spent performing each behavior, and thicknesses of arrows correspond to the likelihood that one behavior would proceed another. The shading ranges from darkest (most time spent performing behavior) to lightest (least time spent). Numerical percentages of time spent in behavior states and transition likelihoods are within circles and beside arrows. Free-swimming ethogram data totaled 10 h of observation, and laser stimulus ethogram observation time totaled 10 min.

The data collected during this analysis were used to create ethograms, which are tools used by behavioral researchers to catalog and visualize an organism’s behavior over an epoch of time (Figure 2). In total, ten hours of free-swimming behavior and ten minutes of laser stimulus behavior were hand-scored using BORIS, an event logging software that allows the user to precisely track different subjects and their behaviors during either live or recorded sessions [18]. The ten hours of free-swimming video data were recorded in blocks of 1–2 h over the course of two weeks during mornings, afternoons, and nights. The 10 min of laser stimulus video data were recorded in roughly 30 s increments during the same two-week period. The behavioral categories were chosen by author S.E. after reviewing the video recordings, in which each distinct behavior performed by the fish was divided into seven categories: swimming (passive movement with no apparent goal), up and down swimming (swimming vertically back and forth at least once), quick swimming (a notable increase in speed), biting (another fish), catching (unique to laser stimulus trials; an obvious and intentional increase in speed culminating in the fish attempting to “catch” the laser dot), foraging (for food and algae in the substrate and on aquarium decorations, aquarium glass, etc.), and hidden. Each action performed by the fish could be labeled using one of the categories described.

## 3. Results

We recorded the responses and color preferences of 66 species of fish. Of these, a great majority (58, or 88%) showed either “some,” “moderate,” or “high” responses to the laser stimuli, with 43 (65%) showing a “moderate” or “high” response to the laser stimuli (see Table 1). Of the three laser colors presented (red, green, and blue), 34 of 66 species (52%) showed an exclusive preferred response to red, 10 (15%) had a response to both red and green, 5 (8%) had a preferred response to red and blue, and six (9%) had a response to red, green, and blue. Only two species had an exclusive preference for green, and only one had an exclusive preference for blue (Figure 1). Supplementary Materials File S1 shows each individual fish species and its color preferences.

The most common behaviors observed upon the presentation of laser stimuli were general attentiveness/tracking, chasing, and catching (whereby the fish would attempt to bite the laser point or catch it in their mouths). These were displayed to varying degrees both within and across species. A list of species by response degree can be seen in Table 1, which shows that the majority of fish were lightly/moderately interested in laser stimuli, displaying obvious but non-extreme behavioral changes upon presentation (i.e., interacting with the laser point for 5 s or less). Eight fish (12%) showed no change in activity during laser stimulus presentation, while 15 fish (23%) were high responders, showing dramatic and instantaneous changes by moving immediately from normal, free-swimming behavior sequences to very intently tracking or chasing the laser dot for five seconds or more. Supplementary Materials File S2 shows a video demonstrating the variety of fish responses to the laser stimuli.

**Figure 3:**
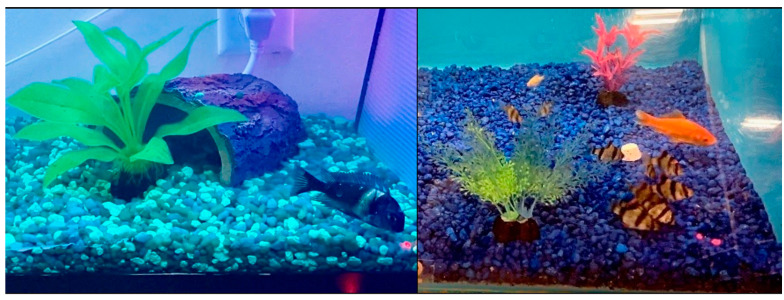
Video stills of a white-spotted cichlid in our home laboratory (**left**) and pet store tiger barbs and goldfish (**right**) orienting towards and chasing after red laser dots. Note that the appearance of two dots in the right hand image is due to reflection of a single laser point on the aquarium glass.

A detailed representation of the “high” response category can be seen in Figure 2, which contains an ethogram comparison of a white-spotted cichlid in our laboratory—the only one of our three white-spotted cichlids who responded to the laser stimuli. In these comparative ethograms, a distinct difference can be seen between those epochs during free-swimming behavior and during laser stimulus presentation. In particular, a dramatic increase in catching and quick swimming behaviors can be seen in the applied laser stimulus ethogram compared with the free-swimming ethogram, and behavioral categories and transitions become much less diverse during laser spot presentation epochs. Additionally, foraging and up and down swimming, which were present during free-swimming epochs, were not present during laser presentation epochs. We include video stills (Figure 3) of our cichlid and pet store tiger barbs, who can all be seen orienting toward and chasing a red laser pointer dot. 

The tiger barbs that were housed in our home laboratory aquarium appeared to show a decreased interest in the laser pointer stimuli (all colors) after about two weeks of shining the lasers into their aquarium multiple times per day, suggesting a response reminiscent of habituation. This decreased interest was indicated by the barbs showing less change in behavior upon the presentation of a laser stimulus than during initial trials. Anecdotally, we also noticed other unusual behaviors during our experiment: (1) goldfish, in general, would not show any change in behavior upon or during the presentation of any laser stimuli. One exception was a goldfish who was housed with the tiger barbs and began to join them in chasing the laser after about 30 s (see Figure 3, Supplementary Materials File S2). (2) Our cichlid—the same cichlid whose behavior is represented in the ethograms (Figure 2 and Figure 3)—would chase after and follow an aquarium plant that was floating on the top of his aquarium when we dragged it around the top of the water. He did not, however, attempt to catch any part of the plant in his mouth as he chased it. (3) Another of our three cichlids would slip through a gap in a barrier intended to separate the fish from one another and spend time in another fish’s section foraging, swimming, and even appearing to tease or incite the other fish to chase him (time spent in the neighboring section was varied and would not always end immediately after being chased). Whether these observations hold behavioral significance and can constitute play is yet unknown.

## 4. Discussion

Can we define the behaviors we observed during the presentation of the laser stimuli as play? We recall Burghardt’s [14] five criteria for what constitutes animal play and apply them to our observations.

1. Chasing, orienting toward, and attempting to catch the laser pointer dots is not likely to be a behavior that contributes to survival, and the repetitive nature of the behavior suggests that it is not intended to serve an immediate function. However, it could also be aggression toward the dot, as it is an unknown stimulus. We note that a cat chasing a laser spot could also be considered an aggressive behavior, but this behavior is considered play [19].
2. The fish were not forced, trained, or enticed to interact with the laser stimuli, proving the voluntary and spontaneous nature of the behavior.
3. The incomplete behavior criterion is perhaps the most difficult to state as being concretely met in our studies. While laser pointer chasing behaviors performed by the fish did appear to be different from those present during displays of aggression toward other fish (behaviors were repeated in quicker conjunction and performed for longer with the laser stimulus), were the fish playing with the laser stimulus, or merely investigating it as being a potential threat or food? Since the interested fish could quickly determine that the laser stimulus was neither food nor a threat but continued to interact with the dot, we can state that this behavior does resemble functional behaviors but may differ in intent. Detailed experiments separating the motivation of the fish regarding the laser pointer stimulus would clarify this point.
4. The observed behaviors were repeated by fish studied throughout multiple trials in our home laboratory. However, loss of interest was observed in our laboratory fish over time, indicating that the behaviors were not pathologically stereotyped.
5. All fish tested were healthy, well-kept, and well-fed, so we can surmise that interactions with laser stimuli were not stress responses.

Our data suggest that the fishes’ reactions to the laser stimulus could be conceived as “play-like” but could also be interpreted as an investigatory response toward a novel stimulus. A novelty response can, however, be considered a key part of play, as parents who give their child a new toy may know. If a fish investigates a novel object that it has never encountered before, is it merely curious (could it be food, could it be noxious, etc.), or is it exhibiting a play response? Our initial results cannot offer a definitive conclusion, but further experimentation potentially could. Creating a way for the fish to intentionally turn on a laser stimulus may allow for more concrete evidence for the presence of play responses. Waterproof buttons that the fish could manipulate with their snouts to turn on a laser pointer stimulus moving about the tank would offer more evidence for self-induced play behavior. Underwater LEDs in various parts of the tank that the fish could turn on and off through their motor behaviors would permit closer examination of the play hypothesis. We had self-righting thermometers in the cichlid tanks in an attempt to replicate previous work by Burghardt et al. [1], but we did not observe our cichlids interacting with them during the 10 weeks we collected data for this project as part of a summer fellowship program for author S.E.

As cats tracking lasers is clearly a form of their predatory behaviors (thus why many cat toys simulate prey), we were curious whether there was any difference between herbivorous and carnivorous/omnivorous fishes’ responses to the laser pointers. Our data set, unfortunately, did not bear this out, as out of 66 of our fish species tested, 63 were carnivores or omnivores. Of the three remaining fish, two species were herbivores: (1) *Pseudotropheus demasoni*—some response to red (a brief change in orientation)—and (2) *Epalzeorhynchos frenatum*—high response to red (behavioral changes for more than 5 s). One species was a “cleaner” fish, *Pseudotropheus crabro*, and had a moderate response to red (up to 5 s tracking). A careful study with higher numbers of herbivores would perhaps show a behavioral difference correlating with laser pointer response, color preference, and diet.

In this work, author S.E. performed all the behavioral scoring, and thus the potential issue of inter-observer variability arises for those attempting to conduct similar experiments. To examine this, 11 individuals from our research group scored the supplemental video (Supplementary Materials File S2) of this work according to our established criteria (none = 0, low = 1, moderate = 2, and high = 3) for the six fishes shown various colored laser stimuli in the video (10 total scoring epochs). We found an average standard deviation of scoring of 0.25, showing that the scoring was relatively consistent between individuals. Most of the variability was in classifying low versus moderate responders. In four epochs that showed clear high responders, the standard deviation among the 11 scorers was zero (meaning everyone choose “3” for the response).

Many videos online show cats, dogs, horses, birds, amphibians, and other fish species reacting to laser pointers. These interactions range from flight to slow chasing to attack. Flight behavior upon laser stimulation can be observed in birds and is even used as such for harvest protection. Nearly all laser attraction behaviors are called playful interactions, perhaps indicative of there being certain play behaviors that function as evolutionary mechanisms that fine-tune innate predation. However, Kogan and Grigg, 2021 [19], showed that cats exhibit an increased risk of abnormal repetitive behaviors during laser play. We observed a wide spectrum of behaviors during fish/laser interactions as well. Some cichlids responded aggressively toward laser pointer stimulation, often seeming to attempt to bite or catch the laser in their mouths, whereas we observed *Devario aequipinnatus* exhibiting an immediate flight response. Some species, such as *Amphiprion ocellaris*, would intently follow and observe the laser, without displaying extreme responses, such as fleeing or aggression. Consequently, our fish play findings reflect the behavioral impact that laser stimulation has on other animal species as well. Further research on fish laser play can elucidate whether the observed behaviors are due to an overstimulation of the visual system that elicits automatic responses, or whether their interactions are indeed playful in nature. In our investigation, we did not observe any abnormal repetitive behavior of the home-lab white-spotted cichlids or tiger barbs that were tested over the course of 10 weeks of this study.

Although 55 of 58 fish that showed responses to lasers displayed a preference for red, the varied difference in response regarding color merits discussion. With increasing depth, long-wavelength red light is more absorbed by water (Figure 1 from [20]), causing an evolutionary shift in species’ spectral sensitivities, e.g., those of Lake Baikal cottoids [21]. The red laser color was revealed as the most salient stimulus for the fish species we studied. A red object that hits the water surface will appear red, but the deeper it sinks, the less and less red light will be reflected. In our investigation, the tiger oscar cichlid (*Astronotus ocellatus*) was the only species that responded solely to blue light stimulation. This species typically feeds on sedentary prey in mud- or sand-bottomed waters [22], where mostly short-wavelength blue light propagates. Consequently, most species that exhibit red light preference live in clear and shallow waters, where color discrimination plays a vital role. The immense variety of species and color sensing cones alone in Malawi cichlids [23] reflects the importance of broad spectral sensitivities in survival [24] and hints at co-evolutionary mechanisms with social interactions, such as mating, territoriality, parental care, and possibly play-like behavior.

We know that mammals and birds play [6], and scientists continue investigating play in the other three classes of vertebrates: amphibians, non-avian reptiles, and fishes. Wrestling and air-bubble riding have been documented in frogs [25], tug of war and precocious sexual play has been documented in turtles [26,27], and object play has been documented in alligators, crocodiles, geckos, and monitor lizards [28,29,30,31]. There are reports that octopuses object-play as well [32,33], and play has been investigated in other invertebrates, such as wasps and spiders [34,35]. Vertebrates diverged from invertebrates approximately 550 million years ago, shortly before the beginning of the Cambrian era, as a subgroup of deuterostomes (chordata). The ancestors of the ray-finned fishes and the ancestors of the tetrapods (from which mammals emerged) separated in the Ordovician period around 450 million years ago. The small number of studies on the evolution of play in other groups of vertebrates, such as fish, do not currently reveal how extended and ancestral play behavior is. The possibility that it emerged prior to the divergence of tetrapods hints at a possible vital role of play in cognitive development [36,37].

## 5. Conclusions

We found that over 80% of the 66 species of aquarium fish tested during this experiment showed a notable interest in moving laser stimuli. While it cannot be definitively concluded that the behaviors at hand were play behaviors proper, they do, when taken together with the currently published literature discussing play in fish [1,2,3,5,6], merit continued experimentation.

Investigations in the cognition of less-understood vertebrates, such as fish, are still in their early stages. Through careful experimental design, observation, and replication by scientists of all ages, sufficient evidence can be gathered to answer the compelling question: do fish play?

## Figures and Tables

**Table 1:** All species of fish tested, according to aquarium labels, by level of interest in laser pointer stimuli (irrespective of color preference). 8 of 66 showed no interest, 15 of 66 showed some interest, 28 of 66 showed moderate interest, and 15 of 66 showed high interest. REG = regular, SM = small, MD = Medium. * The *Devario aequipinnatus* was classified as having high interest in the laser stimuli but did not present with the same or similar behaviors as other fish in this category—rather than simply orienting towards or chasing after the stimuli, all *Devario aequipinnatus* would begin rapidly swimming and darting around their tank immediately upon presentation of the laser stimulus.

No Response 12.1% (8 of 66) *Aulonocara* *Betta splendens* *Carassius auratus* *Carassius stratus species* *Cichlidae (REG assorted)* *Hemigrammus rhodostomus* *Phoxinus phoxinus* *Rocio octofasciata*	Some Response: brief change in swimming direction / orienting towards the laser pointer. 22.7% (15 of 66) *Amphiprion ocellaris* *Astronotus ocellatus* *Aulonocara* *Carassius auratus (2 varieties)* *Cyprinus rubrofuscus* *Elacatinus evelynae* *Epalazeorhychnos frenatum* *Gymnocorymbus ternetzi* *Pethia padamya* *Poecilia reticulata* *Pterophyllum* *Sudotropheus demasoni* *Trichopodus trichopterus* *Siphophorus maculatus*	Moderate Response: following and investigating laser pointer for up to five seconds. 42.4% (28 of 66) *Desmopuntius johorensis* *Amblyglyphidodon aureus* *Chromis viridis* *Cichlidae (SM assorted)* *Cichlidae (MD assorted)* *Coilsa lalia Corydoras panda* *Danio rerio* *Gymnocorymbus ternetzi* *Hyphessobrycon eques (2 varieties)* *Hyphessobrycon flammeus* *Labidochromis cichlid* *Melanochromis johanni* *Nimbochromis venustus Poecilla latipinna* *Poecilla reticulata (4 varieties)* *Pseudotropheus crabro* *Sphaeramia nematoptera* *Tanichthys albonubes Trichogaster lalius* *Xiphophorus maculatus (4 varieties)*	High Response: interacting with the laser pointer for at least five or more seconds. 22.7% (15 of 66) *Amphiprion ocellaris* Devario aequipinnatus * Epalzeorhynchos frenatum *Gramma loreto* *Gymnocorymbus ternetzi* *Melanotaenildae* *Phenacrogrammus interruptus* *Pseucochromis fridmani* *Puntius aurilius* *Puntigrus tetrazona (5 varieties)* *Xiphophorus maculatus*

## Data Availability

The data presented in this study are available in this article and the Supplementary Material.

## Supplementary Materials

The following supporting information can be downloaded at https://www.mdpi.com/article/10.3390/ani12131684/s1, Supplementary Materials File S1 shows each individual fish species and its color preferences, Supplementary Materials File S2 shows video examples of fish behavior in response to the laser stimuli.
