# Supplementary figures and images for: Gills Just Want to Have Fun: Can Fish Play Games, Just like Us?
Source: Animals (Basel). 2022 Jun 30;12(13):1684. doi: 10.3390/ani12131684 (PMC9265024; doi:10.3390/ani12131684)

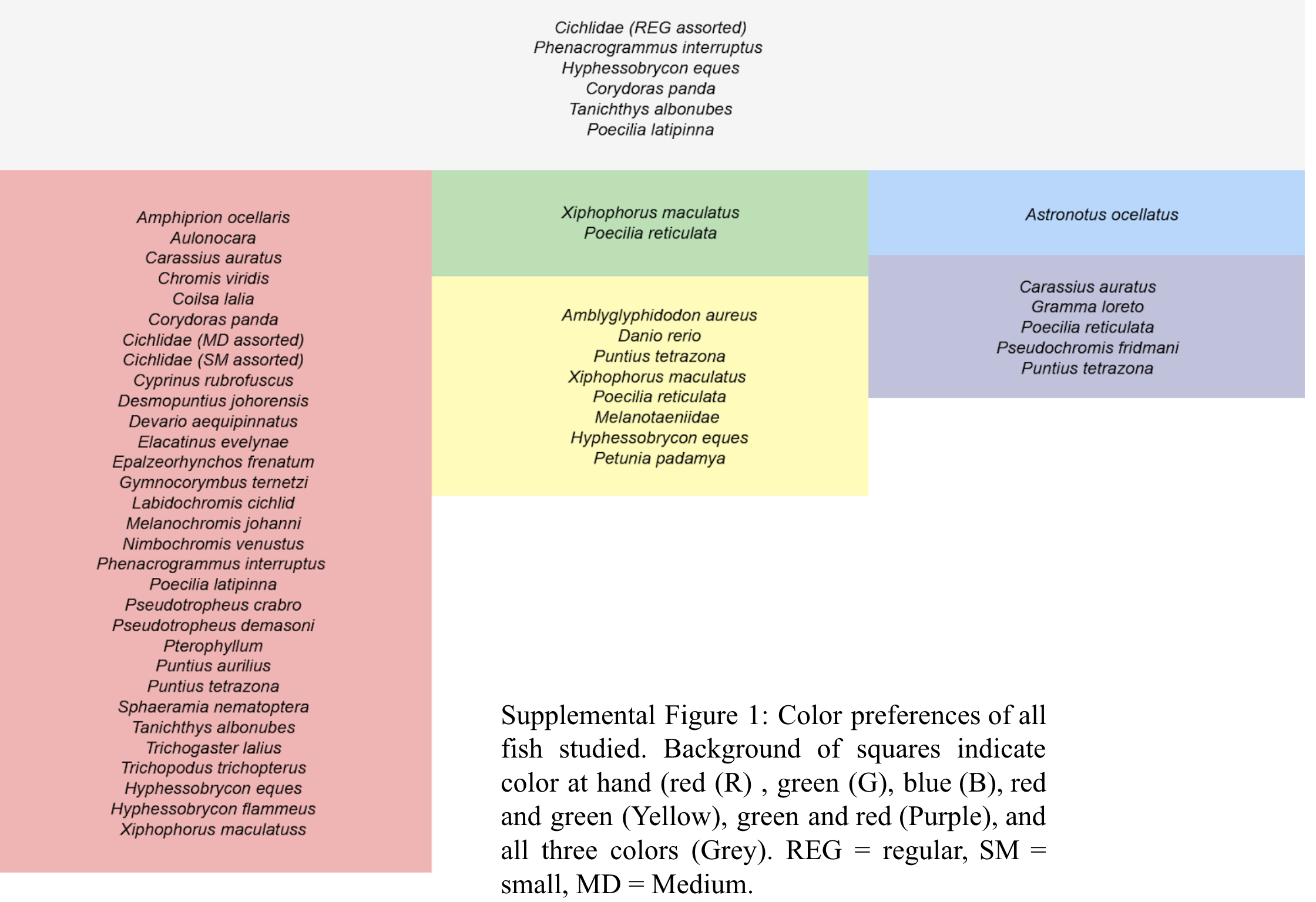

Supplement: Supplementary file 1 [file animals-12-01684-s001.zip › animals-1786860-supplementary/Supplementary Materials File S1 .jpeg]
